# Magnetic resonance myelography in early postoperative lumbar discectomy: An efficient and cost effective modality

**DOI:** 10.4103/0019-5413.65145

**Published:** 2010

**Authors:** Pankaj R Patel, Bharat R Dave, Ujjval H Deliwala, Ajay Krishnan

**Affiliations:** Smt NHL Municipal Medical College and Sheth K M School of Post-graduate Medicine and Research, Ellisbridge, Ahmedabad, India; 1 ‘Stavya’ Spine Hospital, Nr. Nagari Hospital, Mithakhali, Ellisbridge, Ahmedabad, Gujarat - 380 006, India; 2 Government Medical College and Sir T Hospital, Bhavnagar, Gujarat, India

**Keywords:** Discectomy, lumbar disc herniation, magnetic resonance myelography

## Abstract

**Background:**

Magnetic resonance myelography (MRM) after lumbar discectomy is all too often an unrewarding challenge. A constellation of findings are inevitable, and determining their significance is often difficult. MRM is a noninvasive technique that can provide anatomical information about the subarachnoid space. Until now, there is no study reported in literature showing any clinico-radiological correlation of post operative MRM. The objective of this study was to prospectively evaluate the diagnostic effectiveness of MRM for the demonstration of decompression in operated discectomy patients and its correlation with subjective and objective outcome (pain and SLR) in immediate postoperative period.

**Materials and Methods:**

Fifty three patients of single level lumbar disc herniation (LDH) justifying the inclusion criteria were operated for discectomy. All patients underwent MRM on second/third postoperative day. The pain relief and straight leg raise sign improvement was correlated with the postoperative MRM images to group the patients into: A- Subjective Pain relief, SLR improved and MRM image showing myelo regression; B- Subjective Pain relief, SLR improved and MRM image showing no myelo regression; C- No Subjective Pain relief, no SLR improved and MRM image showing myelo regression and; D- No Subjective Pain relief, no SLR improved and MRM image showing no myelo regression.

**Results:**

The result showed that Group A had 46 while Group B, C and Group D had 4, 2 and one patients respectively. Clinico-radiological correlation (Clinically diagnosed patient and findings with MRM correlation) was present in 47 patients (88.68%) which includes both A and D groups. The MRM specificity and sensitivity were 92% and 33.33% respectively.

**Conclusion:**

MRM is a non-invasive, efficient and reliable tool in confirming postoperative decompression in lumbar discectomy patients, especially when economic factors are to be considered and the required expertise to reliably read a complex confusing post-operative MRI is not available readily. Further, controlled double blinded multicentric study in operated and non operated LDH, with MRI comparison would give better evidence to justify its use in screening to detect persisting compression and to document decompression.

## INTRODUCTION

The results of lumbar discectomy after lumbar disc herniation are good, although reherniation rates ranging from 3 to 6% are reported to occur in the early postoperative period.^1^–^3^ Postdiscectomy, MRI studies focus rather on early postoperative changes related to surgery.^4^,^5^ MRI after discectomy if shows an epidural mass or enhancement, then determining their significance is often impossible. During the first few postoperative months the normal signs of scar enhancement, deformity, and mass effect is often misleading.^6^ MRM is a noninvasive technique that can provide anatomical information about the subarachnoid space and can be readily added to a routine MR examination of the spine.^7^,^8^ It helps to understand the MRI images better and increase the level of confidence of the radiologist because of the rapid assessment of the thecal sac and the nerve roots. MRM, in minority of the cases, helps revealing over- or underestimation of nerve root compression compared with MRI alone.^9^ Gadolinium-enhanced MRI is generally considered superior to all other imaging in post discectomy.^10^ MRM has increased the diagnostic yield of the MRI for the detection of foraminal stenosis in cervical spondylotic radiculopathy.^11^

The objective of this study was to prospectively evaluate the diagnostic effectiveness of MRM for the demonstration of decompression in operated discectomy patients and its correlation with subjective and objective outcome (pain and SLR) in immediate postoperative period.

## MATERIALS AND METHODS

This study is a prospective study of 53 patients of LDH operated at a single institute by two operating surgeons from June 2005 to June 2007. The study was approved by Institutional Ethics Committee. The patients aged between 20 and 70 years, single level LDH excluding extra foraminal location requiring surgery, no significant systemic abnormalities (diabetes, neuropathy or disorders requiring DMARD or steroids as treatment), absence of other concomitant spinal diseases, sufficient knowledge of the English or local language to complete the questionnaires were included in the study. Patient not giving informed consent; patients treated with any peri-dural steroids within four weeks or treated with oral steroids, women with a positive pregnancy test with no history of previous surgery on spine and patients with ferromagnetic implants were excluded.

Preoperative analysis with a questionnaire concerning their symptoms, physical examination, MRI and MRM were done by of three surgeons [PRP, BRD, UD] to eliminate any potential treatment bias. The measures that were evaluated included the straight leg raise (SLR) sign and radicular pain.^12^,^13^ In case of radicular pain, the patient was asked to evaluate only leg pain. The pain was evaluated on a standard visual analog scale (VAS)^13^ , on which 0 represented no pain and 10 represented the most excruciating pain imaginable. The patient’s mark was converted to a score: (0) 0 cm = none; 1–3 cm = mild; 4–6cm = moderate; 7–10 cm = severe. The SLR sign, as examined in both legs, and the angle between couch and leg at which the patient experienced pain was recorded. The rating for each leg was converted to a score of 0 to 3 as follows: 0= 90°, 1 = between 75° and 90°, 2=between 45° and 75°, 3= less than 45°. The highest score (smallest angle) was used for analysis.

All patients were operated with discectomy by senior surgeons [PRP, BRD]. All the patients were operated under general anesthesia in prone position through a midline approach by laminectomy/ fenestration and discectomy. Operating surgical loupe of 3× magnification was used. All the patients who were having very tight canal intraoperatively underwent laminectomy. Disc was removed in piecemeals using a pituitary rongeur. No curettes were used intradiscally. Meticulous hemostasis was achieved in all cases. Closure was done over drain. Drain was removed after 24 hours and activities within pain limits were allowed. Patients were allowed to resume their duties within three weeks. Regular physiotherapy protocol (isometric back exercises at three weeks and additional flexion exercises at 8 weeks) was followed. All patients underwent MR Myelography (without complete MRI) on second/third postoperative day.

MRM was performed using turbo spin-echo sequence with extremely long effective TE. The imaging parameters for the lumbar spine, an inversion pulse, were applied to completely suppress the fat signal. In each patient, three images (in coronal and in bilateral oblique coronal directions) were obtained with a slice thickness of 5 mm. T2-weighted MR images allows better anatomic resolution.

Postoperative clinical evaluation was done on second/third postoperative day by the same surgeons, who had done pre operative evaluation. They were evaluated for the SLR sign and radicular pain. The positive change of score by two points was considered significant for radicular pain and SLR sign improvement. Postoperative evaluation of MRM images was done by a reader [AK] who was blinded to the clinical findings and pre operative MR and MRM to avoid bias. The Leg pain relief and SLR sign improvement was correlated with the postoperative MRM images to group the patients into four groups [Table 1].

**Table 1:** Grouping (A, B, C and D) according to postoperative MRM and Clinical findings

MR myelography appearance	Clinical findings leg pain relief (Subjective) & SLR relief (Objective)	
	Present	Absent
Regression	Group A	Group C
No regression	Group B	Group D

Paired sample ‘*t*’ test was used to determine whether there was significant improvement in leg pain and SLR following the operative procedure in the immediate postoperative period. Sensitivity and specificity was used to study the performance of postoperative MRM. Positive was considered to be MRM detecting a state of compression, where as negative was considered to be MRM detecting a state of decompression. For sensitivity and specificity following definitions were used. Sensitivity is percentage of patients who are identified correctly to be in compressed state, where as specificity is percentage of patients who are identified correctly to be in decompressed state. As, preoperative MRM sensitivity was 100% (all patients having compression), ability to detect compression on MRM was used to calculate sensitivity postoperatively also. Due to this the sensitivity of the procedure will come less but it is able to detect decompression better. If the ability of the MRM in detecting decompression (and not compression) is used to calculate the sensitivity, then sensitivity will increase. But same definitions need to be used for preoperative and postoperative MRM findings, thus making it uniform.

## RESULTS

The mean age of the patients were 41.91years (22-68 years) including 32 males and 21 females. Forty two patients were sedentary workers, and 11 were heavy workers. Nineteen patients had presented with history of weight lifting or other straining or twisting activity correlating to symptoms. Most common presentation was backache with sciatica in 45 patients (84.91%), five patients (9.43%) had only sciatica and in three patients (5.66%), backache was more common than sciatica. There was preponderance for left side (*n*=27). The average duration of back pain was 14.6 months and leg pain was seven months in the patients. The average duration of conservative management was less than six months in 36 patients. The pre-operative pain VAS scale score was ‘3’ (severe) in all the 53 patients (the mean VAS scale measurement was 9.34±0.84). The pre-operative SLR score was ‘3’ (*n*=50, 94.33%) and ‘2’ (*n*=3, 5.67%) respectively. A linear correlation between leg pain and SLR was found. Variable neurological motor weakness (toes /foot weakness) was present in 24(45.29%) patients. 16 (30.19%) patients had objective sensory dysaesthesias. Two patients had bladder bowel hesitancy and constipation. The level of involvement in pre-operative MRI was L4-L5 (*n*=22), L5-S1 (*n*=28), L3-L4 (*n*=2) and L2-L3 in one patient. Preoperative MRM showed indentation in 26 patients, complete block in 20 patients, root sleeve block in five patients and double block in two patients (clinically corroborative lower block). Central (*n*=22) and paracentral (*n*=18) position were the most common followed by lateral (*n*=8) and foraminal (*n*=5) location which were characterized as protrusion, extrusion, sequestration in 18, 27 and 8 cases respectively. 28 cases underwent fenestrastion discectomy and 25 cases underwent laminectomy discectomy. Average postoperative hospital stay was five days.

Two patients had temporary retention of urine after the surgery, which was relieved by single catheterization. The postoperative period was uneventful. Dural tears occurred in three patients, which were meticulously closed. The postoperative pain VAS scale score was ‘0’ (*n*=10), ‘1’ (*n*=40) and ‘3’ (*n*=3). The postoperative mean VAS score was 2.19±0.84.

A paired sample ‘*t*’ test showed that the above changes were statistically significant (*P*<0.001), which shows a significant postoperative reduction in subjective patient’s perception of pain. The post-operative SLR score was ‘0’ in 14 patients,‘1’ in 36 patients and ‘3’ in three patients. A paired sample ‘t’ test showed that the objective SLR changes were statistically significant (*P*< 0.001).

The results were grouped as per the clinical finding (leg pain relief/SLR improvement) and postoperative MRM [Table 2].

**Table 2:** The division of patients into groups and their further management

Group	Number of patients (%)	Further postoperative management
A	46 (86.79)	Mobilized regular, with graded physiotherapy
B	4 (7.55)	Mobilized regular, with graded physiotherapy
C	2 (3.77)	Mobilized regular, with graded physiotherapy Analgesics given along with Tablet Duloxetine hydrochloride, counseling and psychotherapy
D	1 (1.89)	Looking to the persistent block, advised MRI and the option of resurgery, but patient refused. Analgesic support continued with activities within pain limit. He responded at 5 weeks clinically and radiologically at 6 months

Early postoperative MRM showed no indentation, block or root sleeve block in 48 patients (Group A+C). In five patients there was block/ indentation detected (Group B and Group D). Fifty patients had significant score improvement of VAS pain and SLR sign. (Group A and Group B)

Preoperative MRM sensitivity was 100% as we included only classical clinico-radiological correlated patients. The postoperative MRM specificity and sensitivity were calculated as 92% and 33.33% respectively.

## DISCUSSIONS

The persistence of pain and functional incapacitation after lumbar disc surgery is especially disheartening within a few weeks or months of surgery and is often accompanied by anxiety, anger, and a great deal of introspection by all concerned. The major causes of failed back surgery syndrome include misdirected surgery (wrong level, wrong diagnosis, unrelated incidental disk herniation, inadequate decompression)^6^ , complications (damaged nerve root, infection, facet fracture), and adverse developments (recurrent herniation, scar formation). Although any treatment should be determined by a specific cause, accurate differentiation is especially crucial when reoperation is considered.^6^

Postoperatively, extensive soft-tissue changes present in the immediate postoperative period severely limits usefulness of MRI. A clear understanding of normal postoperative healing is necessary to avoid overreaction to misleading findings.^6^,^12^ Selection of the initial imaging technique must simplify the diagnostic work-up. In this respect, gadolinium-enhanced MRI is generally considered superior to all other imaging techniques.^10^ The reported accuracy of enhanced CT is 71%, unenhanced MRI 79% and post-gadolinium MRI 96-100%.^6^ MRI is the primary diagnostic tool in lumbar and cervical disc herniation and stenosis because of its high soft tissue discrimination, multiplanner capability, non ionizing, non invasive nature but has suboptimal ability to diagnose foraminal stenosis and osteophytes. MRM helps in the detection of cervical foraminal disease in two ways. First, the heavily T2 weighted nature of the technique resulted in high contrast between the CSF and all other soft tissue structures, including disc material. Second, any possible abnormality seen on MRM prompted a re-evaluation of MRI.^11^

MRM is a recent development in MRI and has advantages in the visualization of the thecal margins, nerve roots and nerve root sheaths.^7^ But, large number of false-positive and false-negative examinations indicates that caution should be used in interpreting MRM.^14^ The diagnostic accuracy of MRM is insufficient to justify its use as an independent diagnostic technique.^15^,^16^ This is attributed to the observation that disc herniation may displace only the epidural fat and not the thecal sac.^14^ MRM provides a high contrast projection image with excellent suppression of background signals justifying its use with conventional MR images to characterize and diagnose specifically, the lesions like congenital abnormalities, traumatic pseudomeningocele, adhesive arachnoiditis, spinal tumors, prolapsed intervertebral disc, degenerative spinal stenosis, AV malformations etc.^17^ Otherwise, there is little evidence to support the use of only MR Myelography in the routine imaging of lumbar degenerative diseases.

**Figure 1:**
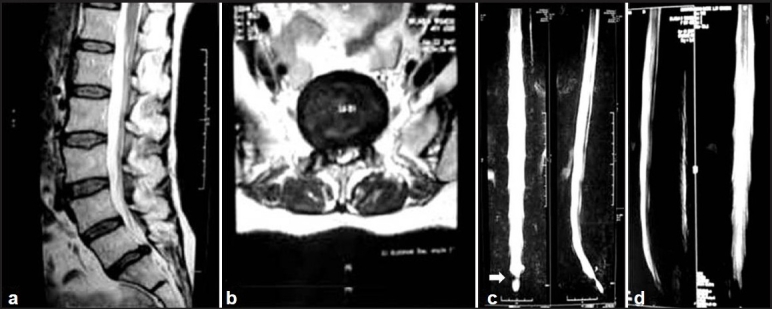
(Group A case): A 50-year-old lady with right paracentral L5-S1 LDH. (a, b) T2 sagittal and axial image (c) Preoperative MRM with block (white arrow) and was then operated right fenestration discectomy. (d) 3^rd^ post-operative day MRM sowing block regression

**Figure 2:**
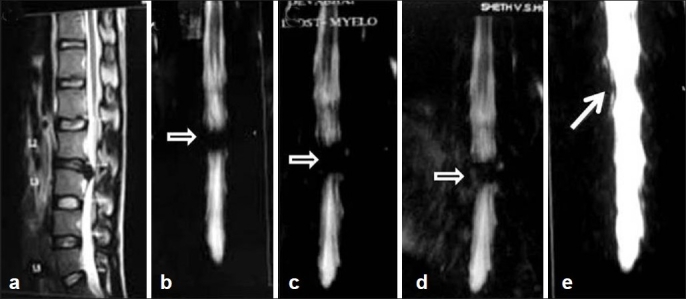
(Group D case): A 29-year-old man with bilateral lower limb radiculopathy (a, b) saggital MRI and preoperative MRM (white outlined black arrow showing block) confirming central herniation and was operated L2 L3 laminectomy and discectomy. (c, d) third postoperative day and sixth week MRM showing persistence of block (white outlined black arrow showing block). (e) Six-month MRM showing resolving block (white arrow)

**Figure 3:**
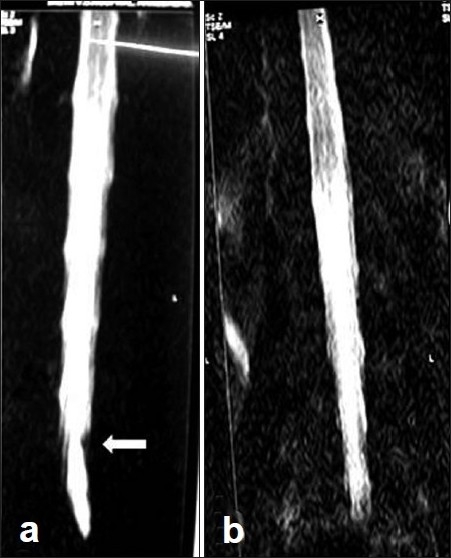
(Group C case): A 38 yr male with clinical left L5-S1 LDH. (a) Preoperative MRM with block (white arrow), was then operated left fenestration discectomy. (b) 3rd post-operative day MRM showing block regression

In this study, patients of LDH are evaluated with the purpose to correlate MRM image with clinical subjective finding of leg pain relief and objective improvement of SLR. Group A patients (*n*=46), very well support adequate surgical decompression [Figure 1]. Group D patients (*n*=1) give some red flags about decompression either adequacy or level [Figure 2]. The only patient in this group was explained MRI and resurgery but, he refused and responded clinically, possibly by natural regression of the remaining disc or the postoperative hematoma at five weeks time. Group C patients (*n*=2) give good confidence to surgeons about decompression at the operated level though no significant pain relief was found initially [Figure 3]. There could be three possibilities in these patients. Firstly, it could be any missed spinal pathology which responded in the mean time. Secondly, root handling and its irritation may result in similar findings at times. Thirdly, a psychological overlap may be present. The two patients of this group gave good response to antipsychotic drugs and on regular follow up there was improvement. These patients could have been identified as poor surgical candidates preoperatively with the Minnesota Multiphase Personality Inventory (MMPI)^18^ or any other better evaluation methodology. MMPI is one of the most reliable and well-documented tests used to predict pre injury susceptibility to back injury and the potential for failure of conservative and surgical treatment. Unfortunately, MMPI is lengthy and difficult to administer in an orthopedic clinical setup.

**Figure 4:**
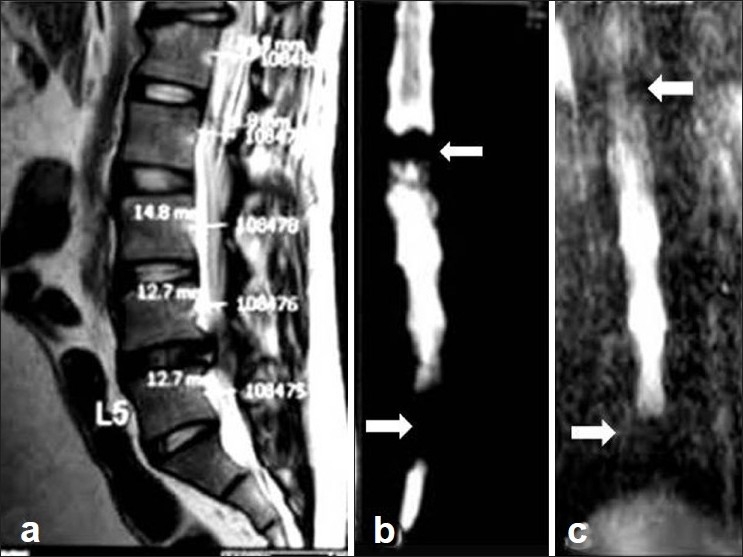
(Group B case): A 34yr old man with right sided signs/symptoms of L4-L5 LDH. (a, b) Preoperative sagittal T2 MRI and MRM showing double level block at L4-5 and D12-L1 (white arrow), but operated for lower level laminectomy and discectomy. (c) Postoperative MRM showing persistence of both block (white arrow)

Group B patients (*n*=4) include patients whose postoperative MRM image remains same as preoperative but had significant clinical improvement. Two of these patients had double level myelography block but clinically symptomatic lower level only was operated [Figure 4]. This technique may be of limited value in patients with multilevel pathology.^15^ The inflammatory properties of the nucleus is well known.^19^ Clinical improvement in these patients may also be explained by washout of the irritant chemicals. Follow up MRM would have given more precise behavior of these block correlations but they were not done. However no long term follow up is available.

So, 47 (88.67%) patients (Group A and D) show correlation of clinical findings supported by MRM image postoperatively. The role of MRM in detecting actual status (decompression or compression: Group A+ Group D) is 88.67%, Sensitivity (ability to detect true compression) and specificity (ability to detect true decompression) of MRM is 33.33% and 92% respectively. The sensitivity written here(33.33%) is the ability to detect compressive lesion/remnant disc/hematoma by MRM. But at the same time the the MRM is able to detect decompression and give surgical adequacy clearance (specificity 92%). Thus, MRM only may be used as screening for surgical adequacy clearance and documentation. Those who have a compressive effect left, may be subjected to detailed MRI for further information and planning. MRI has revolutionized the diagnostic work up of patients with neurologic disorders, but is also leading to additional investigations and rising health care costs.^20^

There could be shortcomings in this study. MRI examination of the patients was not done for control, comparison and addition to the findings. Preoperative and postoperative evaluation was done by the same observers which could have contributed to some bias, but it was attempted to negate the bias with a blinded reader of the postoperative MRM. Still, MR myelography alone had an insufficient diagnostic accuracy to justify its use as an independent imaging technique for the evaluation of LDH. But, in the postoperative period after discectomy MRM is a non-invasive tool to evaluate decompression especially when economic factors are to be considered and the required expertise to read a complex confusing post-operative MRI is not available readily. Further, controlled double blinded multicentric study in operated and non operated LDH, with MRI comparison would give better evidence to justify its use in screening to detect persisting compression and to document decompression.
